# Delinquents to Texas State Medical Association

**Published:** 1885-11

**Authors:** 


### DELINQUENTS TO THE TEXAS STATE MEDICAL ASSOCIATION,

Whose names have been dropped from the roll, are cordially requested to attend the Dallas meeting next April, and be reinstated. We have suggested to the President to recommend in his address that such be reinstated upon the payment of one year's dues—the arrears being remitted; in other words, that they be permitted to regain their membership upon the same terms as new members are admitted. The President has signified his consent, and will so recommend; and there is not a doubt but that such action will be taken, upon his recommendation. It is proposed to hold out every inducement to reputable physicians to unite with the organized branch of the profession. Many are delinquent from having been unable, by reason of sickness or misfortune, or press of business, to attend the meetings. Having been deprived of the benefit of the meetings, it looks hard to make them pay for what they have not had, or to make it harder for them to become members than others, simply from having once been admitted. We advise to wipe out all past indebtedness, as an inducement to members to return. We have the precedent in the American Medical Association, and in the American Public Health Association. ORGANIZATION is the watchword, and THIS JOURNAL is its champion and advocate. "Rally 'round the Flag" of Rational, Ethical, Hippocratic Medicine, and let the profession be purified of pretenders. Mark us—in no other way can the practice ever be regulated, than by organization.

